# Supplementary material for: Reduced isometric knee extensor force following anodal transcranial direct current stimulation of the ipsilateral motor cortex
Source: PLoS One. 2023 Jan 6;18(1):e0280129. doi: 10.1371/journal.pone.0280129 (PMC9821721; doi:10.1371/journal.pone.0280129)
Supplement: Attachment — Submitted filename: Response to reviewers comments[1] Aug 5.docx [file pone.0280129.s001.docx]

When submitting your revision, we need you to address these additional requirements.

1. Please ensure that your manuscript meets PLOS ONE's style requirements, including those for file naming. The PLOS ONE style templates can be found at

<https://journals.plos.org/plosone/s/file?id=wjVg/PLOSOne_formatting_sample_main_body.pdf>  and

<https://journals.plos.org/plosone/s/file?id=ba62/PLOSOne_formatting_sample_title_authors_affiliations.pdf>

**Authors’ response**: The manuscript has been modified in accordance with PLOS ONE style templates.

2. Please provide additional details regarding participant consent. In the ethics statement in the Methods and online submission information, please ensure that you have specified what type you obtained (for instance, written or verbal, and if verbal, how it was documented and witnessed). If your study included minors, state whether you obtained consent from parents or guardians. If the need for consent was waived by the ethics committee, please include this information.

**Authors’ response**: Full ethical information is provided.

3. Thank you for stating the following in the Acknowledgments Section of your manuscript:

“This research was partially funded by the Natural Science and Engineering Research Council of Canada (Grant number 2017-03728). The experiments comply with the current laws of the country in which they were performed. The authors have no conflict of interest to declare. The datasets generated during and/or analyzed during the current study are not publicly available, but are available from the corresponding author who was an organizer of the study.”

We note that you have provided additional information within the Acknowledgements Section that is not currently declared in your Funding Statement. Please note that funding information should not appear in the Acknowledgments section or other areas of your manuscript. We will only publish funding information present in the Funding Statement section of the online submission form.

Please remove any funding-related text from the manuscript and let us know how you would like to update your Funding Statement. Currently, your Funding Statement reads as follows:

“This research was partially funded by the Natural Science and Engineering Research Council of Canada David Behm: RGPIN-2017-0328”

Please include your amended statements within your cover letter; we will change the online submission form on your behalf.

**Authors’ response**: Funding related text has been removed from the manuscript as requested. The following statement regarding funding is fine.

“This research was partially funded by the Natural Science and Engineering Research Council of Canada (David Behm: RGPIN-2017-0328).”

4. We note you have included a table to which you do not refer in the text of your manuscript. Please ensure that you refer to Table 2 in your text; if accepted, production will need this reference to link the reader to the Table.

**Authors response:** In an earlier edition we included more tables. The latest edition only has one table and that is now included in the text.

Reviewers' comments:

Reviewer's Responses to Questions

**Comments to the Author**

5. Review Comments to the Author

**Reviewer #1:**

The manuscript is a technically sound piece of scientific research with data that supports the conclusions. It appears that all appropriate statistical analyses were utilized to reach the authors' conclusions.

**Authors’ response:** Thank-you for the supportive comments.

**Reviewer #2:**

The aim of this study was to determine if anodal transcranial direct current stimulation (a-tDCS) to the motor cortex (M1) modulates quadriceps isometric maximal voluntary contraction (MVC) force or fatigue contralateral or ipsilateral to the stimulation site. Authors main conclusions are that a-tDCS may be ineffective at increasing maximal force or endurance and instead may be detrimental to quadriceps force production.
Because of the rise in popularity of a-tDCS as a tool with potential to influence sport performance, the aims of the present study could be considered relevant to the field. However, the manuscript has some limitations that decreased the initial enthusiasm.
First of all, a single blind approach (with only subjects blinded to condition) could not be the best way to perform this research. Double blind is recommended in this kind of study in which investigators can inadvertently influence the experiment’s results due to hypothesis expectations (i.e., for example, with differences in the way subjects are encouraged during the session).

**Authors’ response**: The reviewer is of course correct that a double blind design is optimal. With the knowledge that the type and intensity of encouragement can influence the participant, we employed a consistent form of encouragement which involved stating “Go, Go, Go” once for each contraction. This information is now inserted into the text as follows:

“All participants were similarly verbally exhorted to maximize each contraction [69], with a consistent form of encouragement, which involved stating “Go, Go, Go” once for each contraction.”

Another question, is the protocol used to induce KE fatigue, which from my point of view does not work well (induced only a 12% decrease in force overall). A time to exhaustion test (TTE) would have been desirable, or at least, a large number of repetitions to increase the perception of effort to a greater extent, since it has previously been argued that modulation of RPE could underpin the tDCS induced beneficial effects on endurance performance.

**Authors’ response:** The fatigue protocol of 12: 5-second MVICs has been incorporated into a large number of published manuscripts from this laboratory. It was chosen since we found it did induce fatigue in these prior studies and the number of published manuscripts using this procedure gave us comparative values if needed.

Below is an incomplete list of some of the studies from our laboratory where we have used the same fatiguing procedure.

*Halperin I,* Ramsay E, Philpott B, Obolski U & Behm DG. The effects of positive and negative verbal feedback on repeated force production**.** Physiology and Behaviour 225: 113086, 2020 DOI: https://doi.org/10.1016/j.physbeh.2020.113086 ^Q1^

Behm DG, Duffett C, Wiseman S, *Halperin I.* Use of Topical Analgesic and Rolling Alone

or in Combination Does Not Increase Flexibility, Pain Pressure Threshold, and Fatigue Endurance — A Repeated-Measures Randomized, Within-Subjects, Exploratory Study Journal of Performance Health Research 2(1):19–26, 2018 DOI: 10.25036/jphr.2018.2.1.behm

Reid JC, Greene RM, Herat N, Hodgson D, Halperin I, Behm DG. Knowledge of repetition range does not affect the maximal force production strategies of adolescent females. Pediatric Exercise Science Journal 29(1): 109-115, 2017 ^Q1^

Halperin I, Copithorne D, Behm DG. Unilateral isometric muscle fatigue decreases force production and activation of contralateral knee extensors but not elbow flexors. Applied Physiology Nutrition and Metabolism 39: 1-7, 2014 ^Q1^

Halperin I, *Aboodarda SJ,* Basset FA, Behm DG. Knowledge of repetitions range affects force production in trained females. Journal of Sports Science and Medicine 13: 736-741, 2014 ^Q1^

Halperin I, *Aboodarda SJ*, and Behm DG. Knee extension fatigue attenuates repeated force production of the elbow flexors. European Journal of Sport Sciences 14(8): 823-829, 2014 DOI 10.1080/17461391.2014.911355 ^Q1^

Halperin I, *Aboodarda SJ*, Basset FA, Byrne JM and Behm DG. Pacing strategies during repeated maximal voluntary contractions. European Journal of Applied Physiology 114(7): 1413-1420, 2014 DOI 10.1007/s00421-014-2872-3 ^Q1^

The other main weaknesses of the study are related to the way it is written. There is a lack of explanations of arguments to support a priori hypothesis and a rather superficial discussion about the results. There are also some inconsistencies between the statistical analysis done and the way results are interpreted and the conclusions raised does not align well with results.

**Authors’ response**: We have revised the manuscript based on the reviewer’s comments and hence we hope it has improved significantly.

Introduction

Introduction is confusing and the rationale is not clear at all. Overall, introduction needs reorganization of content and a better justification of the aims of the study.
For example, there is no proper explanation of the physiological rationale for the effects of a-tdcs over M1 on muscle force. All the justification is based on the effects of a-tdcs on MEP amplitude. Although elucidating the mechanisms of a-tdcs is not the aim of the present study, a proper justification of the selection of stimulating areas, explaining possible physiological mechanisms by which they might influence motor behavior, should be included. Furthermore, it should be better physiologically justified the interaction between both motor cortices and its reciprocal influences, both in terms of facilitation and inhibition, when altering the “excitability” of one hemisphere. The rationale of cross education does not fit well in the intro, or at least at the main argument.

**Authors’ response**: Our apologies but we are confused about the reviewer’s statement indicating that the justification is based only on MEP amplitudes and not on research concerning force changes. In our original version, we cited 19 studies that reported t-DCS induced increases in force and endurance, 15 studies that showed no effect and then described the results of 4 meta-analyses and our prior published narrative review. Hence with 19 studies showing positive results and 15 showing no effect, we feel we covered the force and endurance literature and demonstrated the conflict in the published findings to date.

However, based on the reviewer’s comments we added an additional three studies demonstrating t-DCS induced increases in strength and endurance, with another study showing increases in strength training volume. To highlight the conflict in the literature we have added another 3 studies showing decreases or no effect of t-DCS on force or endurance, and similarly single studies showing no changes in jump, single or repeated sprint performances. We have also added further information to highlight the gaps and conflicts in the literature.

Regarding mechanisms, we have added to the evidence of acute changes in ipsilateral activation by demonstrating the conflicts found with corticospinal excitability with non-local fatigue effects. The additional information is provided below. We have removed the information pertaining to cross education since the present study was an acute study and as the reviewer suggested cross education may not pertain closely to the present argument.

**Examinations of cortical and spinal excitability of the contralateral, non-exercised limb after an acute session of unilateral fatiguing exercise (monitoring of ipsilateral corticospinal influences) have revealed conflicting results with both decreases [49,50] and increases in cortical excitability [51,52] as well as increases [49,50], decreases [51] and no significant change [52] in spinal excitability.** Indeed Cabibel et al. [53] did discover cross facilitation or interhemispheric interactions with tDCS targeting the left M1 inducing both excitatory and inhibitory processing of the left M1. Similarly, a single session of strength training with a-tDCS increased strength, decreased SICI, and increased motor evoked potentials in the contralateral wrist extensor muscles [54]). In their review, Cabibel et al. [55] explained that tDCS-induced cross activation both reduces intracortical inhibition and increases interhemispheric excitatory inputs in the ipsilateral M1 allowing an increase of corticospinal excitability in both upper and lower limbs contributing to motor control. This transfer could result in performance enhancements in muscles ipsilateral to the site of stimulation. Studies have also found evidence of interhemispheric facilitation of the motor cortices using sub-motor threshold intensity TMS stimulation, which suggests the existence of an underlying facilitatory neuronal circuit [56,57]. One study using a single pulse supra-threshold TMS design demonstrated that a-tDCS could increase excitability of the contralateral motor cortex [58].

Lines 137 – 140: This is too speculative Hard to believe that tDCS may exert an effect on the reticulospinal system. My recommendation is to delete this sentence.

**Authors’ response**: We have removed the sentence as requested.

There is a need to actualize the citing literature, because there is a lot of missing references regarding the effects of TDCS on maximal force production and endurance performance. See some of them:

The Effect of Anodal Transcranial Direct Current Stimulation on Quadriceps Maximal Voluntary Contraction, Corticospinal Excitability, and Voluntary Activation Levels.
Kristiansen M, Thomsen MJ, Nørgaard J, Aaes J, Knudsen D, Voigt M.
J Strength Cond Res. 2021 Mar 3. doi: 10.1519/JSC.0000000000003710. Online ahead of print.

Anodal transcranial direct current stimulation enhances strength training volume but not the force-velocity profile.
Alix-Fages C, García-Ramos A, Calderón-Nadal G, Colomer-Poveda D, Romero-Arenas S, Fernández-Del-Olmo M, Márquez G.
Eur J Appl Physiol. 2020 Aug;120(8):1881-1891. doi: 10.1007/s00421-020-04417-2.

Transcranial Direct Current Stimulation Does Not Improve Countermovement Jump Performance in Young Healthy Men.
Romero-Arenas S, Calderón-Nadal G, Alix-Fages C, Jerez-Martínez A, Colomer-Poveda D, Márquez G.
J Strength Cond Res. 2019 Jul 31. doi: 10.1519/JSC.0000000000003242. Online ahead of print.

Transcranial direct current stimulation and repeated sprint ability: No effect on sprint performance or ratings of perceived exertion.
Alix-Fages C, Romero-Arenas S, Calderón-Nadal G, Jerez-Martínez A, Pareja-Blanco F, Colomer-Poveda D, Márquez G, Garcia-Ramos A.
Eur J Sport Sci. 2021 Feb 25:1-10. doi: 10.1080/17461391.2021.1883124. Online ahead of print.

Acute effects of transcranial direct current stimulation on cycling and running performance. A systematic review and meta-analysis.
Shyamali Kaushalya F, Romero-Arenas S, García-Ramos A, Colomer-Poveda D, Marquez G.
Eur J Sport Sci. 2021 Jan 7:1-13. doi: 10.1080/17461391.2020.1856933. Online ahead of print.

Effects of Anodal Transcranial Direct Current Stimulation on Training Volume and Pleasure Responses in the Back Squat Exercise Following a Bench Press.
Rodrigues GM, Machado S, Faria Vieira LA, Ramalho de Oliveira BR, Jesus Abreu MA, Marquez G, Maranhão Neto GA, Lattari E.
J Strength Cond Res. 2021 May 5. doi: 10.1519/JSC.0000000000004054. Online ahead of print.

Transcranial Direct Current Stimulation Does Not Affect Sprint Performance or the Horizontal Force-Velocity Profile.
Alix-Fages C, Garcia-Ramos A, Romero-Arenas S, Nadal GC, Jerez-Martínez A, Colomer-Poveda D, Márquez G.
Res Q Exerc Sport. 2021 Nov 4:1-9. doi: 10.1080/02701367.2021.1893260. Online ahead of print.

**Authors’ response:** We have added this information and references to the manuscript as suggested.

Methods
There are some concerns regarding some questions of the methods and design:

Why authors have chosen a single blind approach (only subjects blinded to condition)? Double blind is needed in this type of research because the authors´ a priory expectation could influence the results of the study (e.g.: via changes in the feedback provided, etc).

**Authors’ response:** The reviewer is of course correct that a double-blind design is optimal. With the knowledge that the type and intensity of encouragement can influence the participant, we employed a consistent form of encouragement which involved stating “Go, Go, Go” once for each contraction. This information is now inserted into the text as follows:

“All participants were similarly verbally exhorted to maximize each contraction [69], with a consistent form of encouragement, which involved stating “Go, Go, Go” once for each contraction.”

The files were anonymized so when analyzing data, the researcher did not know the condition.

For sample size estimation, authors have chosen the effect size based on previous literature (Hazime et al. (2017) and Lattari et al. (2020a)) who found very large effect sizes. However, all the meta-analysis that studied the effects of a-TDCS on motor performance (strength and endurance) found much lower effect sizes (SMD: 0.20 to 0.40). Please justify this decision, and how it would influence the selection of lower ES.

**Authors’ response**: While not all our findings displayed large magnitude effects, there were large magnitude reductions for example in participants’ maximal left (ipsilateral to tDCS) KE force (14%; d=1.09), as well as relative MVC force with a-tDCS (85.92 ± 12.75%), versus s-tDCS (96.18 ± 10.54%) [*t*(15) = -3.07, *p* = 0.016, *d* = -0.77]. Other findings such as the decrease in force from repetition 1 to repetition 12 achieved moderate magnitude effect sizes (d=0.54). Naturally, the power of the statistics would have benefited from a larger sample size, but recruiting significantly more participants was not possible. Hence, the power analysis estimates were applicable to the force changes found in the present study.

A significant interaction effect for condition x leg tested [*F*_(1,56)_ = 8.12, *p* = 0.006, ηp^2^ = 0.13], showed a significantly lower left quadriceps (ipsilateral to tDCS)
Regarding the stimulation site, it has been located at the C3/C4 according to the 10-20 electrode placement system. However, leg representation is located in Cz (just in the sulcus), so it is rather difficult to argue that it is possible to focally stimulate left leg M1 using a 5 x 5 cm anode placed over C3. If we take this into account, the “a priory hypothesis” of this study could not be tested.

**Authors’ response**: Prior studies have used the C3 and C4 locations to stimulate leg muscles. For example, Angius et al. (2018) targeted C3 and C4 and found improvements in cycling performance. Flood et al. (2017) tested endurance of leg extensors while targeting C3 and C4. We also originally cited Montenegro et al. (2015) (knee extensors and flexors) and Vargas et al. (2018) (quadriceps) who used C3 and C4 locations according to the 10-20 Electroencephalography system to stimulate the lower limbs. We have added the Angius and Flood citations in the methods when referring to the C3-C4 site stimulation as follows:

The M1 was located via the C3/C4 locations according to the 10-20 electroencephalography (EEG) electrode placement system [18-20,29,30].

Line 207 -211: Why authors use different protocols before and after the application of tDCS (anodal/sham)? Subjects warmed-up in the PRE but not after 10 minutes of rest (while applying tDCS). Furthermore, they performed 2 to 3 MVCs in the PRE, but only one in the POST. This could influence the results obtained.

**Authors’ response**: The supervising / corresponding author has published over 330 articles and when conducting similar strength and endurance experiments, we have used this procedure hundreds of times. The warm-up consisted of a 5 min cycling routine and three warm-up contractions. Hence, the duration of the warm-up was approximately 7 minutes. If we added a second warm-up to the post-test then testing could not begin for about 7 minutes after the t-DCS. Typically, for testing purposes, there were two MVIC contractions but as mentioned the possibility of 3 MVIC if there was more than 5% difference between the first two contractions. There was no need to repeat this procedure again as the research has shown that the positive effects of warm-up activities on subsequent performance have been reported to be sustained for at least 8 minutes (Wilson et al. 2012, O’Grady et al. 2021), 9 min (Linder et al. 2010), 8-12 min (Kildulff et al. 2007, DeRenne et al. 2010), 10-15 min (Paillard et al. 2018), 18 minutes (Faigenbaum et al. 2010 with adolescents), and 20 minutes (Chiba et al. 2022, Swinta et al. 2022, Tsurubami et al. 2020). Hence, the 10 minutes of tDCS or rest (control) should not have had a negative impact on subsequent performance. We have added this information to the manuscript.

Line 222: please revise the Fatigue Index equation (and the obtained results), it is wrong. It should be as follow:
FI= ((a-b)/a)*100, where “a” is the initial performance and “b” the final performance.

**Authors’ response**: The equation has been changed as suggested.

Discussion

Discussion needs substantial improvement. Overall arguments and explanations exposed by the authors are rather superficial and imprecise. As with the rest of the manuscript, writing needs a thoughtful revision. See some specific comments below:

**Authors’ response:** The discussion has been amended based on the reviewer’s subsequent suggestions.

Line 303: fatigability

**Authors’ response:** According to the Oxford, and Merriam Webster dictionaries (as well as other dictionaries) both modes of spelling (fatiguability or fatigability) are acceptable.

Line 308-3318: I do not understand why it is hypothesized an increase in the MVC while most of the papers who tested 1RM or MVC failed to demonstrate an effect of tDCS on maximal force production, regardless of the muscle tested (please see Alix-Fages et al., 2019). Kristiansen et al., 2021 previously found a lack of increase in MEP amplitude and MVC in a KE MVC after a-tDCS. It should be better addressed and discussed.

**Authors’ response:** As mentioned in the introduction we originally cited 19 research studies that showed an increase in maximal force and endurance. Now with the revisions, we cite 24 studies. Of course, there are others that do not demonstrate increases (14 cited studies). Hence, we highlight this conflict in the literature. Then we cite 4 meta-analyses that show at minimum small positive effects of tDCS on force/strength. However, based on your comment we have revised the sentence in the discussion as follows:

“Since four meta-analyses [43-46] report at least a small positive effect of tDCS on strength, we hypothesized an a-tDCS-induced increase in contralateral KE MVC force. However, this effect was not observed in the present study.”

Line 334-337: This is mere speculation. First, M1 excitability has not been measured. Second, it is not well stablished a causal link between M1 increased excitability and force enhancement. Furthermore, this is not aligned with the main interpretation and conclusion of the paper, which is completely opposed to this result.

**Authors’ response:** Sentence has been removed.

Line 352-354: This is important, because the task could influence results. See Kaushalya et al., who showed that only TTE test are affected by TDCS during running and cycling endurance activities. Furthermore, recent papers found that aTDCS does not influence performance during repeated “all out” efforts (4-6 sec) interpesed with 30 sec rests (i.e.: RSA test: Alix-Fages et al., 2021). Moreover, as previously commented, the task used in this study only produced a 12% reduction in force overall. This should be discussed further.

**Authors’ response**: We have added the suggested information.

Acute effects of transcranial direct current stimulation on cycling and running performance. A systematic review and meta-analysis.
Shyamali Kaushalya F, Romero-Arenas S, García-Ramos A, Colomer-Poveda D, Marquez G.
Eur J Sport Sci. 2021 Jan 7:1-13. doi: 10.1080/17461391.2020.1856933. Online ahead of print

Transcranial direct current stimulation and repeated sprint ability: No effect on sprint performance or ratings of perceived exertion.
Alix-Fages C, Romero-Arenas S, Calderón-Nadal G, Jerez-Martínez A, Pareja-Blanco F, Colomer-Poveda D, Márquez G, Garcia-Ramos A.
Eur J Sport Sci. 2021 Feb 25:1-10. doi: 10.1080/17461391.2021.1883124

Conclusions

“This study found that 10 minutes of 2 mA of a-tDCS is not an effective or consistent method for increasing maximal force production or reducing fatigue in the KE either contralateral or ipsilateral to the stimulated M1.”

This conclusion contrast with those mentioned in lines 334-337.

**Authors’ response**: We have deleted the sentence from lines 334-337 and thus the conclusion aligns with the summary of results in the first paragraph of the discussion.

**Reviewer #3:**

This reviewer is thankful for the opportunity to review this submission by Savoury et al. The aim of the study was to measure the effect of anodal transcranial direct current stimulation (a-tDCS) of the left motor cortex (M1) on both ipsilateral and contralateral knee extension strength and fatigue using a sham-controlled crossover study design. The authors concluded that, contrary to their initial hypothesis, the use of a-tDCS did not significantly increase strength of muscle contraction with contralateral knee extension and significantly decreased muscle contraction with ipsilateral knee extension. They additionally found no significant effect of a-tDCS on muscle fatigue. This work contributes to a growing collection of publications on a-tDCS, all with inconsistent findings regarding its efficacy on muscle contraction augmentation. While the topic is interesting and has promise to translate into clinical space, its findings add to overall ambiguity within the current science. The work is generally well-written, although there is opportunity to provide more clear and consistent use of right/left and contralateral/ipsilateral as shifting between the two sets of terms leads to an unnecessary cognitive burden for the reader. This work provides findings contrary to many published studies, which may be noteworthy, but the authors do not sufficiently reconcile issues they raise in their introduction regarding inconsistency in published tDCS protocol. Consequently, it is difficult to conclude if their contrary findings are a true finding or a result of poorly controlled method with small sample size. With some modifications this work may be fit for publication.

ABSTRACT
Minor Comments
1. Break into sections: Background, Methods, Results, and Conclusion (See Plos One criteria)

**Authors’ response**: Subtitles added as requested.

INTRODUCTION
Major Comments
1. The authors spend a paragraph describing variables that contribute to the efficacy of tDCS. Even when they describe previously used methods in muscle force production and fatigue, they highlight heterogeneity (i.e., contralateral M1 vs temporal cortex vs prefrontal cortex). It is not well resolved what approach might be the most efficacious. Based on what is written, there seems to be insufficient knowledge to support methodological choices.

**Authors’ response**: In the introduction we hoped to highlight the conflicts in the literature. Hence, the reviewer is correct that there is not unanimity regarding protocols contributing to the diverse findings. For our protocol, we were in accord with prior studies that used the C3 and C4 locations to stimulate leg muscles. For example, Angius et al. (2018) targeted C3 and C4 and found improvements in cycling performance. Flood et al. (2017) tested endurance of leg extensors while targeting C3 and C4. We also originally cited Montenegro et al. (2015) (knee extensors and flexors) and Vargas et al. (2018) (quadriceps) who used C3 and C4 locations according to the 10-20 Electroencephalography system to stimulate the lower limbs. We have added the Angius and Flood citations in the methods when referring to the C3-C4 site stimulation as follows:

“The M1 was located via the C3/C4 locations according to the 10-20 electroencephalography (EEG) electrode placement system [18-20,29,30].”

2. Moreover, the introduction is hard to follow, unfocused, and too lengthy. The previous research is presented without flow and how it relates to the current study as well as muscular force specifically. Additionally, much of the details should be reserved for the Discussion Section.

**Authors’ response**: We have removed the section highlighting variables that can influence the administration and effectiveness of tDCS since this was not a factor or a research question in our experiment. We have also removed the information regarding cross education effects as our study examined acute effects.

3. Lines 148-150: Sex differences did not seem to be an active area for investigation. Authors may add background for this or exclude this hypothesis, as no meaningful investigation was conducted to actually observe differences and as of now seems out of place.

**Authors’ response:** As mentioned in the statistical analysis, sex differences were analyzed for relative force changes but no differences were observed. We have now mentioned this directly in the results section.

Minor Comments
1. Line 148: Due TO the lack…

**Authors’ response:** Changed as requested.

2. Throughout the paper, this reviewer suggests using the wording “targeting the left M1” instead of “of the” as it is more reflective of the administration of tDCS

**Authors’ response**: Changed as requested.

3. Line 79-81: If this sentence remains in the article, make wording clearer with more detailed outcomes.

**Authors’ response**: We have provided more clarity to the sentence as follows:

“Similarly, Machado et al. [44] found that tDCS improved muscle strength with 66.7% of parameters tested (e.g., isometric, constant load) compared to only 50% of the parameters tested that demonstrated improvements in muscle endurance (i.e., cycling time to exhaustion).”

4. Issues with sentence structure and clearly presenting the previous literature to introduce the topic to the reader.

**Authors’ response:** We have revised the introduction to provide more clarity and logical progression to the background literature.


METHODS
Major Comments
1. Although the authors refer to Thair et al., 2017 paper for screening, it is important to spell out for the reader in the methods the specific Inclusion/exclusion criteria used to enroll participant.

**Authors’ response**: We have added the following information as suggested.

“Participants were recreationally active with no history of musculoskeletal disorders and were screened for their suitability to receive tDCS based on tDCS checklist recommendations by Thair et al. [61], which included, personal and family history of epilepsy, metal implants, implanted medication pump, pacemakers, recurring headaches, serious head injuries/surgeries, pregnancy, heart disease and various medications.”

2. Works cited in the methods session regarding intervention design are not addressed in the introduction. Why were particular protocols chosen as opposed to others?

**Authors’ response**: We have added the following information to the introduction.

“Therefore, using a similar stimulation protocol as the only other study to test ipsilateral tDCS effects [19] and the recommendations by the Savoury et al. [42] review, the main goal of this study was to determine whether unihemispheric a-tDCS of the M1 is capable of modulating maximal force production or fatiguability of either the contralateral or ipsilateral KE.”

3. The introduction states that this work builds upon the work of Vargas et al. (2018), but this study chose to stimulate and then test versus test at 13 minutes in a 20-mintue stimulation. It is unclear why the authors strayed from this established protocol.

**Authors’ response**: Although Vargas chose to test at 13 minutes within the 20 minute protocol, most other protocols test after the stimulation. In terms of practical relevance, most individuals would not attempt to perform, compete or be tested while being stimulated. Hence for the greatest ecological validity, it was deemed most practical to test after stimulation.

4. Cogiamanian et al. (2007), Kan et al. (2013), Abdelmoula et al. (2016), and Lampropoulou & Nowicky (2013) all use tDCS to measure changes in elbow flexion. The knee and elbow are distinct areas of the motor strip with leg and knee sitting more midline/parasagittal. Montenegro et al. (2015) measured knee flexor, but this was a negative study. Explanation for why this protocol is applicable for your knee flexion study would be helpful.

**Authors’ response**: For our protocol, we were in accord with prior studies that used the C3 and C4 locations to stimulate leg muscles. For example, Angius et al. (2018) targeted C3 and C4 and found improvements in cycling performance. Flood et al. (2017) tested endurance of leg extensors while targeting C3 and C4. We also originally cited Montenegro et al. (2015) (knee extensors and flexors) and Vargas et al. (2018) (quadriceps) who used C3 and C4 locations according to the 10-20 Electroencephalography system to stimulate the lower limbs. We have added the Angius and Flood citations in the methods when referring to the C3-C4 site stimulation as follows:

“The M1 was located via the C3/C4 locations according to the 10-20 electroencephalography (EEG) electrode placement system [18-20,29,30]. a-tDCS protocols had a constant stimulation intensity of 2 milliamps (mA) with a duration of 10 minutes [21,31,33].”

5. It was noted that measurements of the knee joint were acquired as knee angle can affect the isometric MVC, however the angles were not documented.

**Authors’ response:** Angle is now documented.

6. It is understandable that post-tDCS, participants performed only one MVC to minimize the effect on fatigue protocol, but this leads to a comparison of pre-tDCS measurements after 2 -3 attempts that took participant related effort (change of +5%) into account.

**Authors’ response**: The supervising / corresponding author has published over 330 articles and when conducting similar strength and endurance experiments, we have used this procedure hundreds of times. The warm-up consisted of a 5 min cycling routine and three warm-up contractions. Hence, the duration of the warm-up was approximately 7 minutes. If we added a second warm-up to the post-test then testing could not begin for about 7 minutes after the t-DCS. Typically, for testing purposes, there were two MVIC contractions but as mentioned the possibility of 3 MVIC if there was more than 5% difference between the first two contractions. There was no need to repeat this procedure again as the research has shown that the positive effects of warm-up activities on subsequent performance have been reported to be sustained for at least 8 minutes (Wilson et al. 2012, O’Grady et al. 2021), 9 min (Linder et al. 2010), 8-12 min (Kildulff et al. 2007, DeRenne et al. 2010), 10-15 min (Paillard et al. 2018), 18 minutes (Faigenbaum et al. 2010 with adolescents), and 20 minutes (Chiba et al. 2022, Swinta et al. 2022, Tsurubami et al. 2020). Hence, the 10 minutes of tDCS or rest (control) should not have had a negative impact on subsequent performance. We have added this information to the manuscript.

7. Tables mentioned EMG MVC data but were not explicitly stated in the paper; only a strain gauge.

**Authors’ response**: Our apologies for this mistake. These tables were removed in earlier versions and revisions of the paper but we missed that they were still mentioned. They are now removed.

8. Typically blinding of tDCS conditions are implemented in protocols. It does not appear the testing was double-blinded. If so, authors will need to comment on this limitation.

**Authors’ response**: After reviewing the qualitative variables section, we believe we made an error in the results. We did not apply the Bonferroni correction to the Post-hoc Friedmans tests, as we thought this was done in SPSS automatically as it is for ANOVA post hoc tests. We believe that this means the p value for each Wilcoxen test (Friedman post-hoc) should be multiplied by the number of pairwise comparisons that were made following a significant Friedman result. We sincerely apologize for missing this initially. We have now revised this results section and the paragraph in the discussion relating to these results. Hence, based on the correct statistics the blinding was successful. We have removed that section of the discussion and simply stated in the results:

**Supplementary tDCS Questionnaire Data**

Only minor side effects were reported following tDCS, including headache, scalp irritation, tingling, itching, burning sensation, and fatigue. Friedman’s ANOVA revealed no significant differences in these variables between the four protocols (all p values ≥ 0.056) suggesting that participants were sufficiently blinded to which type of stimulation they were receiving.

Minor Comments
1. Experimental design noted to be crossover in abstract. Similar verbiage not used in the body of the manuscript. The authors do say, “repeated measures design, with all participants completing four protocols,” which is the equivalent, but there may be some value to maintain consistency.

**Authors’ response**: we have added the term “crossover” to the first sentence of the experimental design section.

2. Line 183: 10–20 electroencephalography (EEG) electrode placement system.

**Authors’ response**: Done.

3. While testing order was randomized, given the sample size was small, did randomization protocol actually result in equal variances?

**Authors’ response:** Yes, there were equal variances.

4. The Supplementary questionnaire could be improved by asking the participants if they believe they just received the a-tDCS or s-tDCS, but again blinding should be made clearer.

**Authors’ response:** We did not ask or poll the subjects. However as there were no significant differences, it indicates that the blinding was successful and therefore a poll was unnecessary.

5. Authors should consider reviewing sentence structure, as run-on and missing words make the narrative hard to follow.

**Authors’ response:** We have reviewed the sentence structure and ensured there were no run-on sentences or missing words.

RESULTS
Major Comments
1. It would be helpful to report the specific main effects that were not significant

**Authors’ response:** We have now specified the non-significant main effects.

2. Line 282: The title should be more specific. Also, the two paragraphs should be more concise. Moreover, this reviewer does not think this section adds much to the overall aim of the study.

**Authors’ response:** Title has been amended to **Supplementary tDCS Questionnaire Data** as requested. We have moved a number of the statistical details (e.g., [*T* = 105, *z* = -3.41, *p* = 0.001]) to Table 1 to make the section more succinct and easier to read.

As mentioned in response to a prior query:

After reviewing the qualitative variables section, we believe we made an error in the results. We did not apply the Bonferroni correction to the Post-hoc Friedmans tests, as we thought this was done in SPSS automatically as it is for ANOVA post hoc tests. We believe that this means the p value for each Wilcoxen test (Friedman post-hoc) should be multiplied by the number of pairwise comparisons that were made following a significant Friedman result. We sincerely apologize for missing this initially. We have now revised this results section and the paragraph in the discussion relating to these results. Hence, based on the correct statistics the blinding was successful. We have removed that section of the discussion and simply stated in the results:

**Supplementary tDCS Questionnaire Data**

Only minor side effects were reported following tDCS, including headache, scalp irritation, tingling, itching, burning sensation, and fatigue. Friedman’s ANOVA revealed no significant differences in these variables between the four protocols (all p values ≥ 0.056) suggesting that participants were sufficiently blinded to which type of stimulation they were receiving.

3. Tables 1-3: Discrepancy with Tables and reporting. Table 3 is referenced in-text (Line 290), but as Table 1 in Line 300. Tables 1 and 2 (at the end of the manuscript) are not referenced in-text at all and does not have a Table legend.

**Authors’ response:** Our apologies for this discrepancy. Through various stages of writing and revisions we decided to go with figures rather than tables and thus the original Tables 1 and 2 were deleted, but we missed the reference in the text to table 3. Now the text only refers to Table 1.

4. Figures 2-4: Authors should add more details of the figures to captions

**Authors’ response:** We have added the following detail.

Figure 1: Experimental Design

Abbreviations: KE: knee extensors, KF: knee flexors, MVC: maximal voluntary isometric contraction, tDCS: transcranial direct current stimulation

Figure 2: Mean participant absolute right (contralateral) and left (ipsilateral) knee extensor force following anodal transcranial direct current stimulation (a-tDCS) or sham tDCS (s-tDCS). (* denotes statistical significance at *p* ≤ 0.05). Circles denote individual participant data and columns represent mean force values in Newtons.

Figure 3: Participant right (contralateral) and left (ipsilateral) knee extensor force fatigue index following anodal transcranial direct current stimulation (a-tDCS) or sham tDCS (s-tDCS). (* denotes statistical significance at *p* ≤ 0.05). Circles denote individual participant data and columns represent mean force values in Newtons.

Figure 4: Mean participant right (contralateral) and left (ipsilateral) knee extensor (KE) force for each contraction of the fatigue protocol. Asterisk highlights a significant (p<0.0001) decrease in force from repetition #1 to #12. Columns and bars represent mean force values and standard deviations in Newtons.

Minor Comments
5. Define a very good and excellent reliability score outside of parentheses.

**Authors’ response:** We have moved the definitions outside the parentheses and also added more detail re: CV as a % of the mean.

6. Line 262: the results are presented as ipsilateral with a-tDCS then s-tDCS followed by contralateral with s-tDCS then a-tDCS. Would recommend reversing the order of s-tDCS and a-tDCS in the contralateral group so that the ipsilateral and contralateral are presented using a similar convention.

**Authors’ response:** The order of a-tDCS and s-tDCS has been changed in the second sentence to maintain a consistent order of reporting as suggested.

DISCUSSION
Major Comments
1. First paragraph, it is not commonplace to re-report statistics (i.e., the p-values) in the discussion section. This reviewer suggests removing.

**Authors’ response:** All p values in this paragraph have been removed as requested.

2. First paragraph should include specific details of the results. For example, it appears the authors are including both results of maximal force production and muscle fatiguability in “force impairment.” Please rectify.

**Authors’ response:** We have clarified the sentence as follows:

“This study demonstrated significant force impairments (s-tDCS with testing of contralateral KE MVC, and a-tDCS with testing of ipsilateral KE MVC) ) and no significant changes (a-tDCS with testing of the contralateral KE MVC and s-tDCS with testing of the ipsilateral KE MVC) in discrete (single repetition) MVC tests.”

3. Lines 320-321: “a-tDCS is not a consistently effective ergogenic aid when the goal is to increase maximal KE force for a discrete contraction”. Is the variation in outcomes (specifically an increase in maximal KE force) a product of the technology or a product of experimental designs with small sample sizes and numerable uncontrolled, confounding variables? (as stated in the next sentence).

**Authors’ response**: The variability we were referring was inter-individual responses to tDCS. We have added this term to the sentence for clarity. This is similar to the common inter-individual responses associated with post-activation performance enhancement (PAPE) seen in the literature (Behm et al. 2004, Andrews et al. 2016, Chaouachi et al. 2011). It seems some individuals are more sensitive or responsive to tDCS just as some individuals are more responsive to PAPE

Behm D.G., Button D.C., Barbour G., Butt J.C., Young W.B. Conflicting Effects of Fatigue and Potentiation on Voluntary Force. Journal of Strength and Conditioning Research 18(2): 2004

Chaouachi, A., Poulos, N., Abed, F., Turki, O., Brughelli, M., Drinkwater, E. J. Behm, D.G. Volume, intensity and timing of muscle power potentiation are variable. Applied Physiology Nutrition and Metabolism 36(5): 736-747, 2011

Andrews SK., Horodyski JM, Macleod DA; Whitten J, Behm DG. The Interaction of Fatigue and Potentiation Following an Acute Bout of Unilateral Squats. Journal of Sports Science and Medicine 15: 625-632, 2016

4. This reviewer is not sold on the importance of Lines 365-380. These statements can be concise enough to mention in the limitations section (which needs more exploration) due to the fact the participants were not directly asked if they believe they received a-tDCS or s-tDCS.

**Authors’ response**: We have removed the paragraph and incorporated the information into the limitations section which we have copy and pasted in response to query #5.

5. Limitation section needs to be developed. Potentially the comments provided in this review will trigger additional limitations of the study besides the small sample size (e.g., blinding efficacy/expectancy beliefs, other regions of the brain that the authors mention). Other considerations would be the 10-min stimulation, could it be that the length of stimulation inadequate to produce a change?

**Authors’ response**: We have amended and expanded the limitation section as follows:

**Limitations**

It might be suggested that the recruitment of 16 participants in this study involving a more variable intervention might have led to low statistical power even though the power analysis indicated that 8-10 participants should provide sufficient power. Furthermore, as only the KE (quadriceps) were tested, future studies should aim to determine if other muscle groups ipsilateral to the site of stimulation are affected in a similar manner, while also utilizing TMS to determine potential changes in M1 excitability. Finally, as reviewed by Savoury et al. (2021), differences in stimulation protocols (e.g., duration, intensity, electrode location, blinding efficacy, individual expectations and others) make it difficult to make direct comparisons with other studies.

6. In the conclusion section, the authors mention athletes and performance enhancements, but this was not previously mentioned as a potential impetus of this study. This should be rectified.

**Authors’ response**: We have added the following sentence to the first paragraph of the introduction as suggested to provide consistency in our message.

“Since athletes are continually seeking performance enhancements, possible effects of tDCS on exercise and sport performance are important considerations.”

7. Lines 392-392 offers a limitation to only studying quadriceps/KE. This should be moved up to the limitations section. Again, same comments for Lines 392-397 are recommendations to limitations and should be discussed in the Limitations section.

**Authors’ response**: We have moved the suggested sections in the conclusion to the limitation section. See amended Limitations section in response to your query #5.

Minor Comments
1. Restatement of hypothesis on line 308 is hard to follow. Consider rewording to say, “the hypothesis that …” or adding another hyphen in “a-tDCS-induced..”

**Authors’ response**: Based on your comment and another reviewer we have altered the sentence as follows:

“Since four meta-analyses [43-46] report at least a small positive effect of tDCS on strength, we hypothesized an a-tDCS-induced increase in contralateral KE MVC force. However, this effect was not observed in the present study.”

2. Would consider changing work reported on line 317 – MVC is measured not reported.

**Authors’ response**: We changed the sentence as follows:

“Additionally, following a-tDCS in the present study, 4/16 (2 males, 2 females) and 2/16 (2 females) participants experienced increased MVC force in their right and left quadriceps, respectively.”
