# Supplementary material for: Reduced isometric knee extensor force following anodal transcranial direct current stimulation of the ipsilateral motor cortex
Source: PLoS One. 2023 Jan 6;18(1):e0280129. doi: 10.1371/journal.pone.0280129 (PMC9821721; doi:10.1371/journal.pone.0280129)
Supplement: Attachment — Submitted filename: Savoury Nov 30 revised.docx [file pone.0280129.s003.docx]

6. Review Comments to the Author

Reviewer #3: Summary of feedback:
This reviewer thanks the author for addressing most of the previous comments. This reviewer, however, still identified issues in organization, sentence structure, clarity, and references, especially in the Introduction. This reviewer suggest that the authors recruit a few other researchers, outside of the authors, to review and provide feedback. I offer the additional suggestions, although not exhaustive, below.

**Authors’ response:** We thank the reviewer for their contributions to improving the manuscript.

ABSTRACT:
Minor Comments
1. Methods: Label the fatigue protocol.

**Authors’ response:** We have added the descriptor “fatiguing” to clarify that the repetitive MVC protocol was a fatigue protocol. The revision is as follows:

“Additionally, a repetitive MVC fatiguing protocol (12 MVCs with work-rest ratio of 5:10-s) was completed following each tDCS protocol.”

2. Line 44: Replace “impairments” with “reduction” to specify direction of the change.

**Authors’ response**: The term impairment is defined as “diminishment or loss of function or ability.” Hence it does specify the direction of the change. Hence a KE MVC force impairment would be defined as a diminishment or decrease in MVC force function or ability. But if the reviewer likes the word “reduction” better then we have made that change as requested.

INTRODUCTION:
Major Comments
1. The Introduction is too long for the information provided. This reviewer suggests being more succinct in reporting information. For example, Lines 82-98 has unnecessary and repetitive details in which the information can be reduced to about 2 sentences. The authors can reserve some of the details for the discussion.

**Authors’ response**: We have integrated the information in the suggested paragraph to just 4 sentences. We have also reduced the introduction from about 4 to 3 pages.

2. Lines 58-62 should be better linked. For example, use of “however” or some other revision to which it would tell the reader, this is what is known clinically without much discrepancy in the literature, however, when investigating the effects of tDCS on exercise and sport performance there appears to be mixed results. This would be a better transition in

**Authors’ response**: We have attempted to better link the information as follows:

“The effectiveness of tDCS for clinical use has shown positive results involving the treatment of depression, anxiety, schizophrenia, Parkinson’s disease, chronic pain, stroke, and other neural-related problems [2-7]. However, the tDCS research on athletic performance is not as consistent.”

3. There are over 60 references in the Introduction alone, which leads to an indication that the information in the introduction is unfocused. The authors should focus in on only articles specific to their research study. For example, are the effects of tDCS on elbow flexion necessary when the current study focuses on lower limb force and endurance? Are studies that target the dlPFC necessary to compare to a study targeting M1?

**Authors’ response:** Nineteen (19) of the citations are provided for one statement which demonstrated:

“…that tDCS is effective at increasing maximal muscle force and endurance [8-25] as well as strength training volume [26].”

Another two sentences illustrate the expansiveness of the non-significant literature with 15 citations:

“…However, many others report no significant effects or decreases of muscle force or endurance when stimulating the motor cortex [9,21,28-37]. There were also no significant effects on jump height [38], or single or repeated sprint performance [39,40] when stimulating the dorsolateral prefrontal cortex.”

Hence, 34 of the citations are focussed on either the performance improvements or the reductions associated with tDCS. We feel it is necessary to comprehensively cover the literature so the reader is familiar with the disparity and conflicts in the prior publications and what has been examined before.

Based on the reviewer’s comment we have reduced the introduction by approximately a page.

Minor Comments
1. Lines 139-143: use active voice or make line 139-140 its stand-alone sentence after the stated aim.

**Authors’ response:** We have removed the first part of the sentence regarding the type of stimulation protocol. We have moved this information to the methods section.

METHODS:
Minor Comments
1. Lines 185-187: It is unclear why the authors are using 5 study references just say that M1 is located at C3/C4 on EEG placement. Are the authors trying to say something else? If not, this reviewer thinks 1 major reference of an EEG placement paper is sufficient.

**Authors’ response:** We would like to illustrate to the reader that this stimulation protocol has been widely used. Thus, the reader should now have confidence that this protocol is valid and reliable.

2. Line 194: Remove double word.

**Authors’ response:** The second repeated word (i.e., protocol) was removed as suggested.

RESULTS:
Minor Comments:
1. It would be helpful to restate the statistical models for each analysis in the results since the variables of the ANOVA vary.

**Authors’ response:** A 3-way repeated measures ANOVA was employed for all testing.

1. Absolute MVC force
2. Relative MVC force
3. Fatigue test.

Hence stating that it was a 3-way repeated measures ANOVA for each measure would be redundant or repetitive with the information already provided in statistical analysis section and would not provide any greater clarity.

With each interaction in the results, we state the variables tested. Below are just 3 examples:

“…with no significant interaction effects found for condition (condition x time), or leg tested (leg tested x time), nor main effects for condition.”

“A significant [*F*_(1,60)_ =7.156, *p* = 0.010, ηp^2^ = 0.11] interaction effect was found for condition and leg tested (condition x leg tested x time)(Fig 2).”

“A significant interaction effect for condition x leg tested [*F*_(1,56)_ = 8.12, *p* = 0.006, ηp^2^ = 0.13], showed…”

DISCUSSION:
Minor Comments:
1. Line 336-338: Make the sentence more detailed based on the references provided (i.e., #10 and #19). For example, the target of tDCS and MVC, etc in these articles.

**Authors’ response:** We have added the following details as suggested.

“However, a previous studies involving a-tDCS of the contralateral leg motor cortex reported improved foot pinch force for 30 minutes post-a-tDCS [10], as well as increased KE MVC force for 60 minutes after a-tDCS [19].”

2. Line 364: Make the effects on sex more specific to the current studies protocol, parameters and variables and not so general.

**Authors’ response:** Line 364 is the last sentence of the previous paragraph and does not discuss sex differences. However, the following paragraph does discuss sex differences and we have added some greater detail. For example: we have added the following information to that paragraph.

A review by Dedoncker [62] suggested that women demonstrated greater accuracy on cognitive tasks with a-tDCS. A previous study that retrospectively re-analyzed data collected from previous transcranial direct current stimulation studies did not report significant cortical excitability differences between the sexes for a-tDCS. However, the effects of cathodal tDCS on neuroplasticity were greater, lasted longer and demonstrated more inhibition for female participants in comparison to males, suggesting that female participants may experience increased effects of tDCS possibly due to the effects of sex hormones [61].

3. Lines 365-369: Authors should highlight the a-tDCS results first, since it relevant to the current study and then introduce that cathode tDCS generated different results.

**Authors’ response:** We have changed the order as suggested.

4. Line 382: The authors introduced TMS without defining it or providing insight into how TMS may determine potential changes in M1 excitability.

**Authors’ response:** We have added the following explanation:

“Furthermore, as only the KE (quadriceps) were tested, future studies should aim to determine if other muscle groups ipsilateral to the site of stimulation are affected in a similar manner, while also utilizing transcranial magnetic stimulation (TMS) to determine potential changes in corticospinal excitability. When combined with transmastoid electrical stimulation, which activates axons in the spinal cord, effects can be distinguished between cortical and spinal excitability or inhibition.”
