# Supplementary material for: Reduced isometric knee extensor force following anodal transcranial direct current stimulation of the ipsilateral motor cortex
Source: PLoS One. 2023 Jan 6;18(1):e0280129. doi: 10.1371/journal.pone.0280129 (PMC9821721; doi:10.1371/journal.pone.0280129)
Supplement: Attachment — Submitted filename: Response to Reviewer comments.docx [file pone.0280129.s002.docx]

Reviewer #3: Summary of feedback:
This reviewer appreciates the consideration of previous feedback and revision of the manuscript. The manuscript has improved, however, this reviewer found it difficult and time-consuming to identify what changes the authors made. On any subsequent revisions, the authors should include a “tracked changes” version, so that reviewers can easily follow the revisions. This reviewer still found sentence structure, clarity issues, and punctuation errors. Attention to detail is imperative. Lastly, more detail should be included in the methods section as to the measures used to assess MVC.

**Response**: Based on your and the other reviewer comments we hope this revised version will be acceptable.

ABSTRACT:
Minor Comments
1. As mentioned in this Reviewer’s previous comments, there is inconsistent use of right/left and contralateral/ipsilateral as shifting between the two sets of terms leads to an unnecessary cognitive burden for the reader. (Lines 42-47 and throughout the manuscript)

**Response:** We have now emphasized the use of ipsilateral and contralateral rather than left and right limbs or muscles throughout the manuscript.

2. Line 37, was MVC force tested following the a-tDCS and s-tDCS? If so, please specify.

**Response:** As stated in the abstract:

“Knee extensor (KE) MVC force was recorded prior to and following the a-tDCS and s-tDCS protocols. Additionally, a repetitive MVC protocol (12 MVCs with work-rest ratio of 5:10-s) was completed following each tDCS protocol.”

3. Line 40 – remove “The main finding of this study..” and simply say “There was a ….”

**Response:** Done.

4. Line 48 – remove the comma after “Although”

**Response:** Done.

5. Line 50 – remove “Hence”

**Response:** Done.

INTRODUCTION:
Major Comments
1. Third paragraph and fourth paragraph describe positive results and negative results of tDCS, respectively. The third paragraph speaks generally about studies that showed increased muscle force and endurance. The fourth paragraph speaks specifically about tDCS applied to the dorsolateral prefrontal cortex/M1. While the conclusion the authors draw starting on line 77 is likely sound, comparing a general pool of studies to those in a particular area may not be comparing the same phenomenon. The authors discuss this in the final line of paragraph four. If the point to be communicated with the reader is that there is likely heterogeneity in results likely as a result of variation in protocols, this point could likely be made more succinctly.

**Response:** We have added another sentence as suggested to the second last paragraph of the introduction (prior to the objectives and hypothesis paragraph). Based on your suggestion we state:

“Furthermore, the heterogeneity and diversity of overall (both sexes) muscle strength, endurance, and corticospinal excitability findings is likely a result of variation in protocols.”

2. Appreciate the addition of discussion around mechanism and selection of tDCS target added from last version

**Response:** Thank you for the supportive comment.

3. Clear stated aim in final paragraph is helpful - would consider adding ‘left’ prior to M1 in this sentence.

**Response:** Done.

4. The inclusion of sex differences, while appreciated, seems to emerge suddenly. Consider providing some additional context.

**Response:** We have added the following contextual information to the introduction.

“There may be sex differences to consider with tDCS. A previous study reported that cathodal tDCS effects were greater in magnitude and duration for female participants, suggesting that female participants may experience increased effects of tDCS when compared to males [82]. Similarly, a multivariate meta-regression by Dedonker et al. (2016) revealed that women demonstrated greater magnitude responses to tDCS, which might be attributed to sex differences in the precise cortical anatomical locations, cognitive task strategies, as well as hormonal differences affecting brain stimulation. A review by Chinzara et al. [43] suggested that the participants’ heterogeneity in terms of sex and genetic diversity requires consideration. Furthermore, the heterogeneity and diversity of overall (both sexes) muscle strength, endurance and corticospinal excitability findings is likely a result of variation in protocols.”

5. SICI was not introduced previously to line 112.

**Response:** SICI was defined in line 106.

6. The differences in sex was outlined without including why this should be studied, despite the lack of research.

**Response:** We have added the following contextual information to the introduction.

“There may be sex differences to consider with tDCS. A previous study reported that cathodal tDCS effects were greater in magnitude and duration for female participants, suggesting that female participants may experience increased effects of tDCS when compared to males [82]. Similarly, a multivariate meta-regression by Dedonker et al. (2016) revealed that women demonstrated greater magnitude responses to tDCS, which might be attributed to sex differences in the precise cortical anatomical locations, cognitive task strategies, as well as hormonal differences affecting brain stimulation. A review by Chinzara et al. [43] suggested that the participants’ heterogeneity in terms of sex and genetic diversity requires consideration. Furthermore, the heterogeneity and diversity of overall (both sexes) muscle strength, endurance and corticospinal excitability findings is likely a result of variation in protocols.”

Minor comments
1. Errors in punctuation: Lines 64, 70, 72
2. Line 74: prefrontal is generally not hyphenated
3. In line 81, the word “reviews” should be removed and state “meta-analyses” instead.
4. Line 99, a-tDCS was already introduced, and should be used.
5. Line 102: It is unclear to this reader what you mean by “acute studies”. Perhaps, “No studies to date have tested the acute effects of a-tDCS on fatigue/endurance…”?
6. Line 124: Neurones -> neurons (twice)

**Response:** All minor comments revised as suggested.

METHODS:
Minor Comments
1. Appreciate the inclusion of the tDCS Questionnaire in methods. While the results may be in supplementary data, using ‘supplementary’ in the title is confusing. Would consider retitling to “tDCS Blinding Questionnaire”
2. Punctuation Errors: Lines 146, 235
3. Either session or protocol should be used in line 162.
4. Line 182: Consider a different word than “deceptive” – “blinding protocol” would be more appropriate
5. Line 193: use of semi-colon between two and four is unclear. Consider using comma “two, four-second…”

**Response:** All minor comments revised as suggested.

RESULTS:
Minor Comments:
1. Line 243-244 for clarity, consider leading the sentence with “Coefficient of variations …”
2. Line 285: Similar concern about removing supplementary from title, and if truly supplemental, direct readers to the supplemental material within the body of the paragraph
3. Define a very good and excellent reliability score outside of parenthesis.

**Response**: All minor comments revised as suggested.

DISCUSSION:
Major Comments:
1. There is opportunity to discuss more about the gender differences and recommendations for future tests based on why gender was included as exploratory in this study. The introduction referred to a lack of literature, however the discussion noted a previous study. Additionally, in the conclusion that there is no difference between males and females is perhaps overstated. Was the experiment actually powered to observe this? Agree that it is worth noting, but that a recommendation for further research with a specific aim on sex differences is warranted.

**Response:** We have expanded the discussion paragraph on sex differences as well as adding a sentence in the limitations section regarding a possible lack of statistical power to detect sex differences.

2. Line 312: you reiterate the point, “this lack of reliability and high variability in the literature may be also related to the great diversity of implemented protocols (e.g., differences in electrode location, size, number, current density, polarity, and stimulation duration) [42].” after reading the introduction and discussion I am still left wondering how your study attempts to address this. Ideally stronger commentary should be provided as to why your method is optimal and why others should continue to apply this method in future studies.

**Response:** Our methods were based on the prior review recommendations of Savoury et al. Based on the variability in the literature and inconsistent results in the present study there may not be an overall “optimal” method. As discussed in greater detail in the discussion in this revised version, there are sex differences in responses as well as interindividual responses. We previously stated:

“This inter-individual variability likely contributed to some of the non-significant results of this study, and in combination with previous research suggests that a-tDCS is not a consistently effective ergogenic aid when the goal is to increase maximal KE force for a discrete contraction.”

We have added the following sentence based on your suggestion:

“These inconsistent results occurred even though the present study implemented the a-tDCS stimulation recommendations of the Savoury et al. [42] review for the methodological variables that were most likely to produce the greatest exercise performance (i.e., muscle strength, endurance) enhancement.”

3. Addition of expanded limitations section is appreciated

**Response**: We appreciate the supportive comment.

4. Lines 350-353 should be included in the introduction as well.

**Response:** This information has been moved to the introduction as suggested.

5. Final sentence of your last paragraph again noted heterogeneity in protocol - what is your recommendation?

**Response:** We have added the following recommendation:

“A series of comprehensive studies are needed to determine optimal stimulation protocols suitable for various populations (e.g., possible differences between sexes, age), specific performance enhancement (e.g., strength, endurance, or cognitive) and more research targeting areas other than the M1 (e.g., temporal cortex and prefrontal cortex).”
